# al-BERT: a semi-supervised denoising technique for disease prediction

**DOI:** 10.1186/s12911-024-02528-w

**Published:** 2024-05-16

**Authors:** Yun-Chien Tseng, Chuan-Wei Kuo, Wen-Chih Peng, Chih-Chieh Hung

**Affiliations:** 1 Department of Computer Science, National Yang Ming Chiao Tung University, University Road, Hsinchiu, 30010 Taiwan; 2 Department of Management Information Systems, National Chung Hsing University, Xingda Rd, Taichung, 40227 Taiwan

**Keywords:** Disease prediction, Semi-supervised learning, Attention, Health insurance data, Electronic medical records, EMR

## Abstract

**Background:**

Medical records are a valuable source for understanding patient health conditions. Doctors often use these records to assess health without solely depending on time-consuming and complex examinations. However, these records may not always be directly relevant to a patient’s current health issue. For instance, information about common colds may not be relevant to a more specific health condition. While experienced doctors can effectively navigate through unnecessary details in medical records, this excess information presents a challenge for machine learning models in predicting diseases electronically. To address this, we have developed ‘al-BERT’, a new disease prediction model that leverages the BERT framework. This model is designed to identify crucial information from medical records and use it to predict diseases. ‘al-BERT’ operates on the principle that the structure of sentences in diagnostic records is similar to regular linguistic patterns. However, just as stuttering in speech can introduce ‘noise’ or irrelevant information, similar issues can arise in written records, complicating model training. To overcome this, ‘al-BERT’ incorporates a semi-supervised layer that filters out irrelevant data from patient visitation records. This process aims to refine the data, resulting in more reliable indicators for disease correlations and enhancing the model’s predictive accuracy and utility in medical diagnostics.

**Method:**

To discern noise diseases within patient records, especially those resembling influenza-like illnesses, our approach employs a customized semi-supervised learning algorithm equipped with a focused attention mechanism. This mechanism is specifically calibrated to enhance the model’s sensitivity to chronic conditions while concurrently distilling salient features from patient records, thereby augmenting the predictive accuracy and utility of the model in clinical settings. We evaluate the performance of al-BERT using real-world health insurance data provided by Taiwan’s National Health Insurance.

**Result:**

In our study, we evaluated our model against two others: one based on BERT that uses complete disease records, and another variant that includes extra filtering techniques. Our findings show that models incorporating filtering mechanisms typically perform better than those using the entire, unfiltered dataset. Our approach resulted in improved outcomes across several key measures: AUC-ROC (an indicator of a model’s ability to distinguish between classes), precision (the accuracy of positive predictions), recall (the model’s ability to find all relevant cases), and overall accuracy. Most notably, our model showed a 15% improvement in recall compared to the current best-performing method in the field of disease prediction.

**Conclusion:**

The conducted ablation study affirms the advantages of our attention mechanism and underscores the crucial role of the selection module within al-BERT.

## Introduction

Electronic medical records (EMRs) serve as a detailed ‘log’ of a patient’s health, encompassing a wide range of data including clinical diagnoses, treatment regimes, test outcomes, and patient-reported information. This wealth of data offers immense potential for deep learning applications in medical analysis. For instance, in assessing diabetes, the analysis of fundus retinal images has emerged as a highly effective tool. Utilizing deep learning models, these images are scrutinized to detect signs of diabetic retinopathy, a critical step in the early identification and management of diabetes and its associated complications. EMRs also play a pivotal role in the exploration of disease interrelations, providing a vast repository of data that enables researchers to delve into patient medical histories and the evolution of diseases, thereby uncovering possible links and patterns among various health conditions. Despite these benefits, the application of EMRs in forecasting present and future health states encounters a myriad of challenges.

In this paper, we study the research problem of predicting a set of diseases that a patient may encounter over a forthcoming period, based on their electronic medical records. Solving this research problem of predicting future diseases from electronic medical records can offer numerous benefits across various aspects of healthcare and public health. Beyond merely considering electronic medical records as basic medical documentation, an interesting approach is to view them as a form of language. Med-BERT [1] demonstrates that when disease records are transformed into sequences, it exhibit similar structures to sentences. However, when transitioning this model to our real-world EMRs, it demands some considerations. First, unpredictable diseases cannot represent a patient’s characteristics. These diseases could be a noise in medical data, making prediction models give an improper result. For example, diseases in external causes of injury are not predictable, and such medical records could be noise for a model. Second, some diseases are very widespread, affecting patients of any age and in any geographic area. These types of diseases cannot adequately represent an individual patient’s situation. Take influenza-like diseases as an example. These diseases typically spread widely during specific seasons, which limits their ability to accurately learn a patient’s overall health condition. Such characteristics in EMR might lead the trained disease prediction model to achieve impressive scores, but might result in lower practical utility in real-world applications.

To overcome this problem, we developed a semi-supervised attention learning method. The attention mechanism operates as a semi-supervised learning feature to identify redundant diseases, capture patient characteristics, and output concise extracted information. Moreover, before activating the attention mechanism, we constructed a graph based on disease relations extracted from the electronic medical records for disease embedding. This graph is built on the principles of comorbidity and disease proximity [2], which imbues the resulting embeddings with medical significance. We combine the attention mechanism with BERT, the pre-trained model with the masked language model task, then fine-tuned our model with both masked language tasks and next sentence prediction tasks.

Since our model combines semi-supervised **a**ttention **l**earning, graph embedding and pre-trained BERT, we named it al-BERT. The model is inspired by the behavior of influenza-like disease in the Taiwanese administrative medical database. Filtering out noise information to obtain concise patient data can aid in more accurate predictions. According to Marsden-Haug [3], influenza-like disease includes 28 diseases in the International Classification of Disease, Ninth Revision (ICD-9)^1^.

The contributions of this paper can be summarized as follows:

- We propose a BERT-based model, al-BERT, which integrates a semi-supervised learning module with the attention mechanism to capture specific information. The learning module mimics the disease assessment process performed by domain experts, employing both Backward RNN and Bi-directional RNN to extract information from the input data.
- Our model combines domain knowledge, such as comorbidity and the distance between diseases, during the training process. The concept involves constructing graph embeddings prior to modeling, enabling us to attain more interpretable embedding results.
- To identify the location of noise diseases, we employ a semi-supervised attention learning mechanism. We label specific segments as noise diseases, and then extract the results based on the model’s learning outcomes.
- The al-BERT model is evaluated on a real-world dataset, which includes 18.2% of influenza-like disease cases that we consider as noise in the disease sequence. By leveraging the attention mechanism, our model aims to filter out the noise and focus on the crucial disease information for accurate prediction.

## Related work

### Machine learning on medical datasets

Machine learning is a powerful tool that can be applied to medical datasets to make predictions, automate processes, and improve medical quality. It involves the use of algorithms to analyze and learn from data, enabling the creation of models that can make predictions or perform tasks without being explicitly programmed to do so. Medical datasets often contain large amounts of complex and heterogeneous data, making them well-suited for machine learning applications.

Different medical data refer to the information collected and recorded about a patient’s health and medical history. This can include demographic information, such as age, gender, and race; personal and family medical history; lab test results; imaging studies; medication and treatment history; and notes and observations made by healthcare providers during patient encounters. Popular tasks include: readmission [4, 5] length of staying days [6, 7], medical recommendation [8–10], drug-drug interaction (DDI) [11, 12], and disease detection [13, 14].

Existing works usually apply multiple types of medical information to enhance the model.

**G-BERT** [8] considers medical ontology to strengthen medical prediction. Medical codes such as drugs or diseases contain natural hierarchical structures, which are hard to observe in patient health records. G-BERT uses ICD9 ontology for diagnosis and ATC ontology for medication. Ontology trees help to embed different codes in records. These codes were applied to BERT, then combine a visit embedding for an input record. G-BERT applies ontology information to enhance embedding quality.

**RETAIN** [15] considers diagnosis, medication, and procedure information for disease prediction. This information is encoded into an input sequence, an is then embedded into a vector. RETAIN applies attention reversely to pay more attention to recent visits.

**CONAN** [13] is a model for rare disease detection. Rare disease here indicates diseases that are individually rare but collectively common. These diseases are easily misdiagnosised due to the lack of content information about the patient. CONAN considers medical codes, diagnosis codes, and procedures to construct a network, then applies GAN with complementary pattern augmentation.

**Dipole** [16] is a paper that presents a novel approach to healthcare diagnosis prediction from historical Electronic Health Records (EHRs). The authors propose using attention-based bidirectional recurrent neural networks (RNNs) to analyze patient data and predict potential medical diagnoses. Attention mechanisms in Dipole measure relationships between visits, enabling effective result interpretation. Experimental results on real-world EHR datasets demonstrate that Dipole significantly improves prediction accuracy compared to state-of-the-art methods, and offers clinically meaningful interpretations, advancing the field of diagnosis prediction in healthcare.

**MMGL** [17] employs modality-aware representation learning to aggregate features from each modality, leveraging both the correlation and complementarity between them. Instead of manual graph definition, the latent graph structure is captured through an effective adaptive graph learning approach. This enables joint optimization with the prediction model, thereby unveiling intrinsic connections among samples.

**MedSkim** [18] addresses the challenge of health risk prediction using electronic health record (EHR) data. Existing approaches often struggle with noisy EHR data. To address this, the authors introduce MedSkim, a novel model designed to automatically remove irrelevant visits and codes from EHR data, thereby improving prediction performance. MedSkim employs a code selection module to skip irrelevant diagnosis codes, a backward probing RNN for coarse-grained representation learning of visits, and a forward skipping RNN to dynamically select important visits and codes. The risk prediction module utilizes the output of the forward skipping RNN for final predictions. The model also includes a regularization term based on skip rate, and combines it with cross-entropy loss for end-to-end training.

Considerations of cross columns or cross datasets are common. Problems can be solved by having more information, but it is more complicated to construct a suitable network for models.

### Language model on medical datasets

Medical records behave similarly to natural language. Therefore, natural language has been used in disease prediction for the analysis of patients’ medical records, symptom descriptions, and other relevant data to identify potential diseases and make predictions.

**Graph Convolutional Transformer (GCT)** [19] extends Transformer [20] to construct a graph from medical data, then applies graph convolutional networks. GCT captures structural information in most types of health records, even those which did not provide a structural relation. They also construct a probability matrix in which each element is defined by the conditional probability of previous records. This matrix helps guide self-attention to learn the structure in medical data.

**med2vec** [21] is a work applying the skip-gram method to capture the visit representation of health records. A similar strategy is used in word2vec [22]. Medical codes are transferred to a binary vector, and are then combined into a visit representation according to patient records. Med2Vec incorporates both code co-occurrence information and visit information of the health records. Applying the skip-gram method and combining co-occurrence and visit information help improve the accuracy of both code and visit representations. Also, they provide a state-of-the-art medical embedding method.

**Cui2vec** [23] is a deep learning model that represents medical concepts in a vector form. It works by mapping all the medical concepts into a common concept unique identifier space using the thesaurus from the Unified Medical Language System (UMLS). Differing with med2vec, cui2vec embeds medical codes into vectors based on words. Besides disease and drugs, medical related journals and clinical notes can also be transferred to vectors by cui2vec. Cui2vec provides a scalable and flexible way of representing medical concepts in a compact and meaningful form.

**Med-BERT** [1] is a method which extends BERT. The authours apply patient visit records to train their model. Each patient’s visit record is considered as a sentence, and medical codes are considered as words. Visit records are arranged to formulate a BERT readable structure without additional datasets and external models. Med-BERT is trained by the masked language model and the next sentence prediction, and is fine-tuned by the masked language model. The fine-tuning and experiment result shows that the meaning of diseases can be learned well by the contextual structure of the health records.

**CoQUAD** [24] is a question-answering system designed for efficient extraction of answers related to COVID-19 questions. Focused on text-based tasks, CoQUAD includes two datasets: a reference-standard dataset derived from CORD-19 and LitCOVID, and a gold-standard dataset curated by public health experts. It is trained on the BM25 algorithm to search the reference-standard dataset for relevant documents based on COVID-19-related questions. Additionally, it features a Reader component consisting of the Transformer-based model MPNet, which reads paragraphs and extracts answers from retrieved documents. In general, CoQUAD performs extraction after the question-answer process, aiming to obtain a more concise answer.

## Preliminary

This section introduces essential definitions and outline the problem statement that will be further explored and addressed in the later sections.

**Fig. 1:**
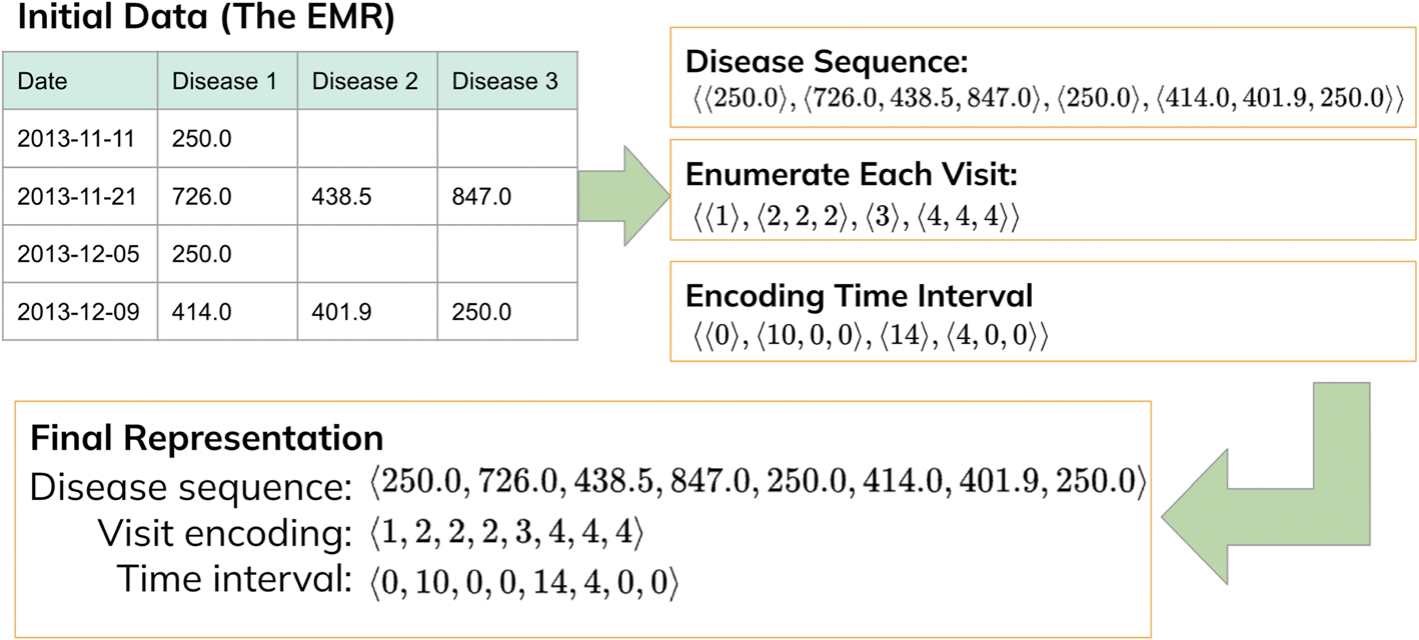
An example of how we transfer an electronic medical record from a patient from tabular data to a sequence. For the disease sequence, we extract each diagnosis record and list them in chronological order. For visit enumeration, we enumerate each visit sequentially. For the time interval, we set the first diagnosed disease as 0 and then calculate the day interval between each diagnosed disease and the previous one

### Definition 1

(Electronic medical records). Let $EMR_p = \langle EMR_{p,1}, EMR_{p,2}, \ldots , EMR_{p,m} \rangle$ represent the electronic medical records of a patient $p$, where each $EMR_{p,i}$ is a tuple $(dt_i, d_{i,1}, d_{i,2}, \cdots , d_{i,m_i})$. Here, $dt_i$ is the date of the $i$-th visit, $d_{i,j}$ is the disease code of the $j$-th diagnosis on date $dt_i$ and $m_i$ represents the maximum number of diseases present in any diagnosis, a value that can vary across different datasets. We can also represent $EMR_p$ into *EMR* if we are not emphasizing a specific patient *p*.

For example, given the example in Fig. 1, the electronic records of this patient is represented as $\langle (\text {2013-11-11},250.0,\epsilon ,\epsilon ),(\text {2013-11-21},726.0,438.5,847.0), (\text {2013-12-05},250.0,\epsilon ,\epsilon ),(\text {2013-12-09},414.0,401.9,250.0)\rangle$.

### Definition 2

(Disease Sequence). Given $EMR = \langle EMR_{1},EMR_{2}, \ldots , EMR_{m} \rangle$ where $EMR_{i}=(dt_i, d_{i,1},d_{i,2},\cdots ,d_{i,m_i})$, the disease sequence is defined as $D = \langle d_{i,j} \mid 1 \le i \le n, 1 \le j \le m_i, d_{i,j} \ne \epsilon \rangle$. Without ambiguity, we denote $D=\left\langle d_1, d_2, ..., d_n \right\rangle$.

The disease sequence extracts the order of disease codes occurring in electronic medical records, which can help us to understand the temporal relationship of disease occurrences. Following the same example, the electronic records in Fig. 1 is $\langle (\text {2013-11-11},250.0,\epsilon ,\epsilon ),(\text {2013-11-21},726.0,438.5,847.0),(\text {2013-12-05},250.0,\epsilon ,\epsilon ), (\text {2013-12-09},414.0,401.9,250.0)\rangle$. The disease sequence can be derived as: $\langle 250.0,726.0,438.5,847.0,250.0,414.0,401.9,250.0 \rangle$.

### Definition 3

(Visit Sequence). Given $EMR = \langle EMR_{1},EMR_{2}, \ldots , EMR_{m} \rangle$ where $EMR_{i}=(dt_i, d_{i,1},d_{i,2},\cdots ,d_{i,m_i})$. The visit sequence is define as $VS = \langle VS_{1},VS_{2},...,VS_{m} \rangle$ where $VS_{i}=\{i^k| k = argmax_j \{d_{i,j}\ne \epsilon \}\}$.

Follow Fig. 1 again. The first visit has only one diagnosed disease, so the positional enumeration for this visit is $\langle 1 \rangle$. The second visit has three diagnosed diseases, so it is $\langle 2,2,2 \rangle$. The third one has one diagnosed disease, hence $\langle 3 \rangle$. The fourth one has three, so hence $\langle 4,4,4 \rangle$. Therefore, our visit sequence is $VS=\langle 1,2,2,2,3,4,4,4\rangle$.

### Definition 4

(Time Interval Sequence). Given $EMR = \langle EMR_{1},EMR_{2}, \ldots , EMR_{m} \rangle$ where $EMR_{i}=(dt_i, d_{i,1},d_{i,2},\cdots ,d_{i,m_i})$. The time interval sequence is define as $TIS= \langle TIS_{1},TIS_{2},...,TIS_{m} \rangle$ where $TIS_{i}=(dt_i-dt_{i-1},0,...,0)$. For the convenience of computation, we set $dt_{0}=dt_{1}$.

Consider the example given in Fig. 1. We have $dt_1 = \text {2013-11-11}$, $dt_2 = \text {2013-11-21}$, $dt_3 = \text {2013-12-05}$ and $dt_4 = \text {2013-12-09}$. For the first visit, we set $\langle 0 \rangle$. For the second visit, since the time interval compared to the previous visit is 10 days, and there are three diagnosed diseases, we set 10 corresponding to the first disease, and 0 corresponding to the following two diseases. Hence, we have $\langle 10, 0, 0 \rangle$. Similarly, the third visit has $\langle 14 \rangle$ and the fourth visit has $\langle 4,0,0 \rangle$. Therefore, our final time interval sequence is $TIS = \langle 0,10,0,0,14,4,0,0 \rangle$.

The visit sequences and the time interval sequences will be utilized to derive disease embeddings in BERT later on.

### Definition 5

(Noise Disease Set). The noise disease set of a disease *d* is a set of diseases $S=\{ s_1, s_2, \cdots , s_m\}$ which are relatively not important to *d*.

Noise disease set is usually chosen by the domain expert and aims to reduce the significance in predicting the comobilities of given the disease *d*. For example, influenza cases are usually seasonal and more likely to be influenced by the environment compared to other diseases. Therefore, influenza cases are perfectly suited to be considered as noise diseases. On the other hand, from patients’ electronic medical records, we can find the comorbidity of a disease by generalizing Valderas’ definition of comorbidity in the medical field [25].

### Definition 6

(Comorbidity). Given a trigger disease $d_{trigger}$, a target disease $d_{\text {target}}$ is a comorbidity of $d_{\text {trigger}}$ if $d_{target}\ne d_{trigger}$ and $d_{\text {target}}$ manifests after $d_{\text {trigger}}$ has occurred $k$ times. Here, the parameter $k$ varies depending on specific conditions within different datasets.

The trigger disease here refers to the disease we are going to observe its comorbidities, that is, its target diseases. It is also worth mentioning that a trigger disease can be associated with multiple target diseases. Consider the example in Fig. 1. Let $d_{trigger}=250.0$ and $k=2$. The disease 401.9 is recognized as the comorbidity of 250.0 since 401.9 manifests after 250.0 has occurred in 2013-11-11 and 2013-12-05. Theoretically, we can designate any disease as a trigger disease if we choose to include its comorbidity in our model. In practice, not all diseases need to be considered as trigger diseases in practice, since some conditions, like appendicitis, are known not to be linked to other diseases.

Building on the definition of comorbidities, we can construct a comorbidity graph to explore the relationships among them.

### Definition 7

(Comorbidity Graph). A comorbidity graph is defined as an undirected graph $G = (V, E)$, where each vertex $v \in V$ represents a disease that appears either in the set of trigger diseases $D_{\text {trigger}}$ or their corresponding target diseases $D_{\text {target}}$. An edge $(u, v) \in E$ exists if disease $u$ is a target disease of $v$ or $v$ is a target disease of $u$. The weight of an edge (*u*, *v*) is $wt(u,v)=\frac{1}{N_{uv}}\sum _{k=1}^{N_{uv}}|R_{u,k}-R_{v,k}|F_{k}$ where $N_{uv}$ is the number of common comorbidities of $u$ and $v$, $R_{i,j}$ denotes the rank of disease $i$ in the frequent comorbidity list of disease $j$, and $F_{k}$ is the Shannon entropy of disease $k$^2^.

**Fig. 2:**
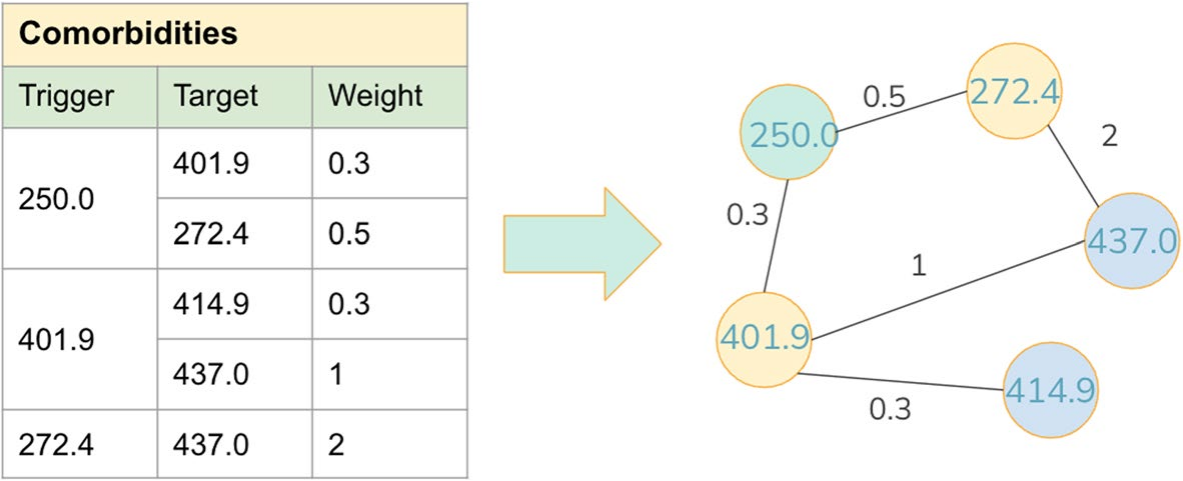
This figure shows a toy example demonstrating the construction of a comorbidity graph for our model. The comorbidity graph is built based on identified comorbidities. Each node in the graph represents a disease, and a link is established between pairs of comorbidity diseases

Figure 2 illustrates the process of constructing a comorbidity graph. Consider three trigger diseases: 250.0, 401.9, and 272.4, with their corresponding target diseases being {401.9, 272.4}, {414.9, 437.0}, and {437.0}, respectively. Since 401.9 is a target disease of 250.0, an edge $(250.0, 401.9)$ exists in the comorbidity graph. Similarly, 401.9 serves as the trigger for target disease 414.9, resulting in an edge $(401.9, 414.9)$ in the graph. In this manner, the entire comorbidity graph is constructed, as shown in Fig. 2. In this work, the trigger diseases are selected by the approach outlined in Yang’s work [27]. A disease that appears more than twice in a single patient’s record and is identified as having comorbidities is selected as a trigger disease. To minimize redundancy, we exclude noise diseases and those diseases that occur in less than 1% of patients.

Having outlined the necessary definitions, we now introduce the research problem in this paper.

### Problem 1

(Disease Prediction Problem). Let *EMR* be the electronic medical records of a patient, *S* be a noise disease set, *G* be the comorbidity graph, and *T* be a time duration. The goal is to predict the diseases being diagnosed over a future period of length *T*.

This section ends with Table 1 for the convenience of the readers. This table provides a list of the symbols and parameters used throughout this paper.

**Table 1:** Symbols used in this paper

Symbol	Description
*EMR*	An electronic medical record from a patient
$EMR_i$	The ith visit of a patient’s EMR
$dt_i$	The record date of the ith visit $EMR_i$
$d_{i,j}$	The jth recorded disease of ith visit
*D*	The disease sequence of a patient consist of diseases
$d_k$	The kth element in *D*
*VS*	The visit sequence of a patient consists of visit enumerations
$VS_k$	The kth element in *VS*
*TIS*	The time interval sequence of a patient consists of time intervals with respect to the previous diseases
$TIS_k$	The kth element in *TIS*
*S*	Noise disease set. Unimportant diseases identified by domain experts in our study
$d_{trigger}$	Trigger disease. The disease we have selected and for which we wish to find closely related diseases
$d_{target}$	Target disease. The diseases closely related to the selected trigger disease
*G*	A graph, specifically referring to the comorbidity graph in our work
*X*	The embedded diseases
$x_i$	A vector in *X* representing the embedding vector of disease $d_k$
*k*	A specified variable we use to determine when to insert the [SEP] token
*T*	A specific time slot that we want to focus on for prediction
$\text {[SEP]}$	A token located between $d_{trigger}$ and $d_{target}$
$X'$	An extracted disease sequence, where $X'\subset X$
$\alpha , \beta$	Adjustable parameter

## Method

### Overview

**Fig. 3:**
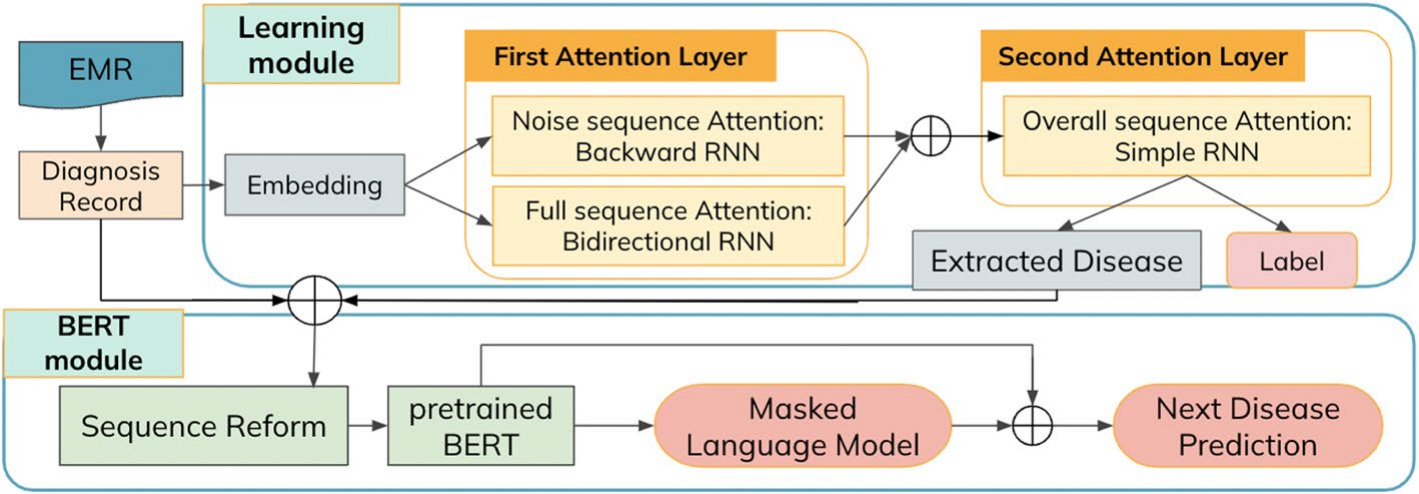
The framework of our work. Our model, al-BERT, is named after attention learning and BERT. al-BERT comprises two primary sections: the semi-supervised learning attention module and the BERT module. The red blocks indicate the output of this model, with the “label” block assisting in training the attention weights. al-BERT processes the input disease sequence through a double attention layer to capture crucial information from the sequence, and then employs the BERT model to predict the outcome of future disease

Figure 3 shows the overview of *al-BERT*, the proposed method. al-BERT contains two modules: the learning module and the BERT module. The learning module adopts semi-supervised attention learning mechanisms. The attention mechanism is applied twice to the embedded disease sequence: one iteration is focused on denoising, while the other is directed towards capturing overall attention. Denoised attention reads the input disease backwards to capture the diseases in noise disease set *S*, and full attention learns characteristics for the input disease sequence. We combine the results, then apply attention to learn the adjusted results for all of the diseases in the record. The disease sequence after the second attention layer can be transferred to weights of the diseases sequence. We set a threshold to extract essential diseases to formulate the patient’s characteristics, then input selected diseases to the BERT module.

The inspiration for the learning module stems from the need to reduce influenza-related data within the disease records, because influenza provides limited information for disease prediction problems. MedSkim [1] similarly utilizes this concept, but it skims through all disease records, whereas al-BERT selectively targets and reduces the number of diseases for advanced analysis. In this paper, we specifically focus on diseases related to influenza-like disease, and we reference these influenza-related diseases using codes from [3].

We integrate the concept of comorbidity into our model by constructing a disease graph that captures the relationships between diseases. A comorbidity graph provides insights into these relationships, enabling us to utilize it for graph embedding to generate an embedding vector for each disease. Additionally, we incorporate comorbidity information into the BERT module by determining the next sentence label based on identified comorbidities. Importantly, this comorbidity information is extracted directly from the dataset, eliminating the reliance on external data sources. By conducting an in-depth analysis of disease co-occurrence patterns within the dataset, we are able to accurately identify comorbidities associated with specific diseases.

### Learning module: attention mechanism

We apply the semi-supervised attention mechanism to capture the importance of disease sequences. Our approach involves modifying the attention mechanism to include two layers. The first layer comprises two parallel attention mechanisms. It is tasked with learning the overall patterns within the disease sequences and identifying the diseases in the noise disease set *S*. The attention mechanism for overall patterns applies bidirectional RNN, while the component responsible for noise reduction operates reverse RNN. These outputs are then passed through a softmax function. The output from the first layer is then used as input for the second attention layer. In the second layer, another round of attention is performed based on the results obtained from the first layer. The second attention layer outputs the weight and label of the disease sequence, providing valuable information regarding the importance and relevance of each disease in the sequence.

The first attention layer consists of two attention blocks: the full sequence attention block and the noise reduction attention block.

- **Full Sequence Attention block**: This block utilizes a bidirectional recurrent neural network to capture the influence of each disease in the entire sequence. The bidirectional recurrent neural network allows us to consider both the forward and backward contexts of the diseases, enabling a comprehensive understanding of their dependencies and relationships within the sequence.
- **Noise Reduction** **Attention block**: This block is designed to focus on learning the importance of a subsequence of the original disease sequence. To achieve this, we employ a reverse recurrent neural network that takes into account the temporal order of the diseases. By considering the sequential information, the model can effectively capture the relative importance of each disease based on its position in the sequence.

The combination of these two attention blocks enables our model to capture both the global influence of diseases in the entire sequence and the specific importance of the selected disease, enhancing its ability to make accurate predictions.

To form our learning module mathematically, consider a disease sequence $\langle d_1,d_2,\cdots ,d_n \rangle$. Each disease $d_k$ can be embedded into a vector $x_k$. These embedded disease vectors, $X = \langle x_1, x_2, \cdots , x_n \rangle$, are then input into the two attention blocks to capture the attention weights and labels associated with the disease sequence.

1 $$\begin{aligned} \begin{array}{l} h_t = \sigma _1 (W_t h_{t-1} + W x_t + b)\\ \hat{h}_t = \sigma _1 (\hat{W}_t \hat{h}_{t-1} + \hat{W}_x x_t + \hat{b})\\ y_t = \sigma _2(W_y h_t + b_y)\\ \hat{y}_t = \sigma _2(\hat{W}_y \hat{h}_t + \hat{b}_y) \end{array} \end{aligned}$$

In the above equation, *W* is the embedding matrix. $W_t, \hat{W}_t \in \mathbb {R}^{p\times q}$, $b, \hat{b} \in \mathbb {R}^p$ , $W_y, \hat{W}_y \in \mathbb {R}^{q \times q}$ and $b_y, \hat{b}_y \in \mathbb {R}^q$ are parameters to learn. $\sigma _1$ and $\sigma _2$ are a non-linear activation function. In our case, we choose the sigmoid function $\sigma _1$ and the hyperbolic tangent function $\sigma _2$. For simplicity, let us denote $h_t$ as the hidden layer of the full attention block, and $\hat{h}_t$ as the hidden layer of the noise attention block.

The outputs from the two blocks in the first layer of attention are combined using the adjustable parameters $\alpha$ and $\beta$. These parameters are adjusted based on the conditional probability of diagnosing trigger disease, diagnosing target diseases, and influenza-like disease. $\alpha$ represents the likelihood of diagnosing the trigger diseases in general, while $\beta$ signifies the likelihood of diagnosing the trigger disease specifically when the patient has a prior diagnosis of influenza-like disease. This combination is represented as:

2 $$\begin{aligned} v_t = \alpha h_t + \beta \hat{h}_t \end{aligned}$$

The second attention layer follows the original Transformer straightforward:

3 $$\begin{aligned} Attention(Q,K,V)=softmax\left( \frac{QK^\intercal }{\sqrt{dim_k}}\right) V \end{aligned}$$

After applying the second attention layer, the attention vector is obtained for each position in the sequence. This attention vector, denoted as $a = (a_1, a_2, \cdots , a_n)$, represents the importance or relevance score assigned to each disease in the sequence. By considering these attention scores, we can evaluate the significance of each disease and use them in subsequent steps, such as disease prediction or further analysis.

To evaluate the prediction label, we apply attention vector *a* with learnable matrix $W'$ and parameter *b*.

4 $$\begin{aligned} y = Sigmoid(W' \cdot a + b) \hat{y} = \sum \limits _i=1 ^{n} y_i \end{aligned}$$

Overall, the learning module in al-BERT plays a crucial role in focusing on specific parts of the input disease sequence. The first attention layer, encoded by a recurrent neural network (RNN), captures the overall patterns in the full disease sequence and the noise diseases, resulting in new disease vectors denoted as $v_t$. The second attention layer operates on these new disease vectors and calculates attention weights for each disease. These two layers are combined then passed through a second attention layer to obtain the final disease representation vector. This module enables our model to effectively capture the essential information and dependencies within the disease sequence, facilitating accurate disease prediction and analysis.

### Extracted patient records

To extract information from the attention layer, we utilize the learned attention scores $(a_1, a_2, \ldots , a_n)$. We introduce a threshold $\theta \in (0,1)$ and define a dense function $f_s$ for the context vector. If the value of $f_s(a_i)$ exceeds the threshold $\theta$, it indicates that the disease $d_i$ is considered important and is included in a new disease sequence $D'$. Each disease in $D'$ is included in *D*, i.e., $D' \subset D$. If the disease is filtered out, we pad 0 in the end of sequence to keep the disease sequence the same length. By applying this thresholding mechanism, we can filter out less relevant diseases and focus on the diseases that have a higher impact or significance in the context of disease prediction.

### BERT module: training

The BERT module leverages BERT to understand the contextual relationships among diseases from given disease sequences. By treating each disease as a word and a sequence of diseases as a sentence, we can borrow BERT to discern the sequential relationships between diseases in a manner akin to how BERT comprehends relationships within sentences. Med-BERT is one of the work demonstrated this concept [1]. However, numerous noise diseases - which are less critical for disease prediction - often appear in disease sequences. These noise diseases can reduce the accuracy of disease prediction, as they disrupt the contextual understanding of diseases much like stuttered words in a sentence. Consequently, it is crucial to identify and remove these noise diseases from the sequences. A significant challenge arises because it is difficult for domain experts to enumerate all noise diseases associated with the given trigger diseases. To address this, we have developed a semi-supervised approach to identify potential noise diseases using a small labeled set of noise diseases, denoted as *S*. Details of this approach will be discussed later in this section. Once noise diseases have been identified and excluded in the learning module, we expect that our input sequence in the BERT module will be more coherent and refined compared to the original sequence. In the BERT module, we utilize a transformer architecture similar to that described in the original BERT paper by Devlin et al. [28]. We also adopt pretraining techniques similar to those used in the original BERT model. Our model, referred to as al-BERT, is pre-trained using two tasks: the masked language model and next sentence prediction. These tasks enhance the model’s ability to understand and generate meaningful disease predictions. For the disease sequence $D'$, which is the selected result after the attention module, al-BERT performs token embedding, segment embedding, and positional embedding, similar to how BERT operates. In detail, token embedding is applied to the disease sequence *D*, segment embedding to the visit sequence *VS*, and positional embedding to the time interval sequence *TIS*.

#### The masked language model

The techniques used in the masked language model task draw inspiration from BERT [28] and Med-BERT [1]. The task involves randomly masking certain input disease tokens, akin to Cloze tasks in the literature. When a position is masked, there is a 70% probability of it being replaced by a [MASK] token, a 15% probability of being replaced by a random code, and a 15% probability of remaining unchanged. This approach aids the model in learning disease characteristics within the context of previous and current diagnosed diseases, enhancing its ability to make accurate predictions. Additionally, it helps the model understand the significance of each disease statement within a disease sequence.

#### Next sentence prediction

The Next Sentence Prediction (NSP) task is one of the two pre-training objectives used in the original design of the BERT model. The NSP task is given pairs of sentences as input and must predict whether the second sentence is the true subsequent sentence that follows the first sentence in the original document, or if it is a random sentence from the corpus. Here, the [SEP] token, short for separator, is used in BERT to mark different segments or sequences within a single input. In the case of NSP, where BERT must handle two distinct sentences to determine if they are sequentially related, [SEP] is used to clearly indicate where one sentence ends and the next begins.

In al-BERT, we use the trigger disease and target disease to separate the disease sequences for training. For each input disease sequence, we identify a length *k* that includes the trigger disease in the sequence. Following the appearance of the trigger disease, we insert a [SEP] token after the visit sequence containing the trigger disease. If any of the diseases in the target set occur after the visits where we input the [SEP] token, we label this pair as the next sentence.

**Fig. 4:**
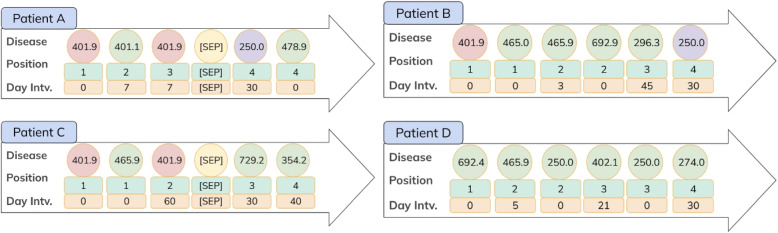
This figure demonstrates the placement of the [SEP] token and the process of setting next sentence prediction labels. The trigger disease is highlighted in red, while the diseases in the target disease set are shown in purple, and the other disease are shown in green

Figure 4 shows an illustrative example of the next sentence prediction task in al-BERT. We selected the trigger disease as 401.9 (Hypertension), chose the target disease as 250.0 (Diabetes mellitus without mention of complication), and set the trigger length k=2. Essentially, this configuration aims to ascertain the potential diseases that could occur in a patient who has been diagnosed with diabetes. For patient A, we inserted a [SEP] token after 401.9, its corresponding visit, and time interval, which represent the trigger disease occurring twice, in order to examine the targets. Since 250.0 occurred after [SEP], the label for patient A is set to 1. The same configuration applies to patient C, but in this case, the target 250.0 did not occur after [SEP], resulting in a label of 0. If a patient has been detected with either trigger or target diseases, but the count of trigger occurrences is less than k, we still append a [SEP] token to the end of the disease sequence and set the label to 0. This ensures that even if there is insufficient trigger information, the patient is included in the analysis while not affecting the label for the target disease. However, in cases where a patient has never been recorded with any trigger or target diseases, like patient D, we exclude that patient from the analysis.

The selection of the trigger disease and target disease set is based on the concept of comorbidity in diseases [25] and Dr. Yang’s study on the distance between diseases using rank order statistics [2]. This choice allows al-BERT to learn the associations and relationships between the trigger disease and the target disease set, enabling it to better understand the context and make predictions about future diseases based on a given disease.

In the pre-training stage of al-BERT, when selecting disease sequences for the next sentence prediction task, we assign labels to 20% of the data as the next sentence and the remaining 80% as not the next sentence. The setting of 20% and 80% is due to the imbalance in the occurrence of comorbidity cases in the dataset.

Specifically, for the 20% of records labeled as “the next sentence”, the disease sequence includes the trigger disease ($d_{trigger}$), and our target disease ($d_{target}$) occurs after the trigger disease in the sequence. These records represent the positive examples for the next sentence prediction task. On the other hand, for 80% of the cases where patient records have been labeled as “not the next sentence”, the disease sequence either includes the trigger disease $d_{trigger}$ occurring under the setting of trigger occurrences *k*, or $d_{trigger}$ happens more than *k* times but the target disease $d_{target}$ did not occur.

By training al-BERT with both positive and negative examples, it learns to understand the relationships between the trigger disease and the target disease set, enhancing its ability to predict future diseases based on a given disease in the downstream prediction tasks.

### BERT module: fine-tuning

In fine-tuning al-BERT, our focus centers on the Next Sentence Prediction (NSP) task, which aims to predict the subsequent disease in a patient’s medical history based on comorbidity relationships, as detailed in “Next sentence prediction” section. This task closely aligns with real-world clinical scenarios, enabling al-BERT to anticipate complex disease sequences, including those arising from comorbidity patterns.

Our fine-tuning process optimizes al-BERT for this NSP task, harnessing the knowledge within the dataset to accurately predict evolving disease sequences. This equips al-BERT to contribute significantly to predictive healthcare analytics, enhancing disease management and patient care.

### Downstream tasks for al-BERT

Our adaptation of al-BERT builds on the Med-BERT architecture, introducing a double attention module before the input of disease sequences into the BERT framework. This module enhances al-BERT’s ability to capture intricate disease relationships and contextual information, significantly improving its predictive capabilities.

To make al-BERT adaptable to downstream tasks, a classification layer or prediction head is added atop the pretrained model, following a similar approach as with Med-BERT. During the fine-tuning process, the parallel double attention layer and prediction head are attached to the al-BERT architecture. Parameters of al-BERT are loaded and initialized from the pretrained model, and both al-BERT parameters and prediction head parameters are updated using gradient descent. Our primary downstream task for al-BERT involves disease prediction within electronic medical records (EMRs), with a specific emphasis on the NHI-CD dataset. This task is geared towards predicting the next potential disease in a patient’s medical history based on their past disease records. Such a task holds immense significance in healthcare analytics, as it empowers early disease detection, proactive healthcare management, and personalized treatment strategies. To initiate the model’s processing, we input a disease sequence into the model. The learning module diligently filters out redundant diseases, and subsequently, the BERT module undertakes the task of predicting whether the patient is likely to develop the target disease in the future based on this refined sequence. This refined sequence helps enhance the fluency of predictions and contributes to the model’s overall effectiveness in disease prediction within EMRs.

## Experiments

The study’s objective is to forecast forthcoming diseases using prior diagnosis records. The process involves learning and mitigating the impact of inconsequential diseases, verifying the presence of particular diseases (trigger), and predicting whether the patient is likely to develop related (target) diseases in the future.

### Dataset

We evaluate al-BERT on two datasets: MIMIC-III [29] and Ambulatory care expenditures by visits (CD) from the Taiwan National Health Insurance (NHI) Research Database (Table 2).

**Table 2:** Statistics of the data

Items	MIMIC-III v1.4	NHI-CD-R301
patients	46,520	39,807
total visits	58,976	7,728,574
diagnosis	651,047	10,907,751
time interval length	10 years	17 years

#### NHI-CD

**Fig. 5:**
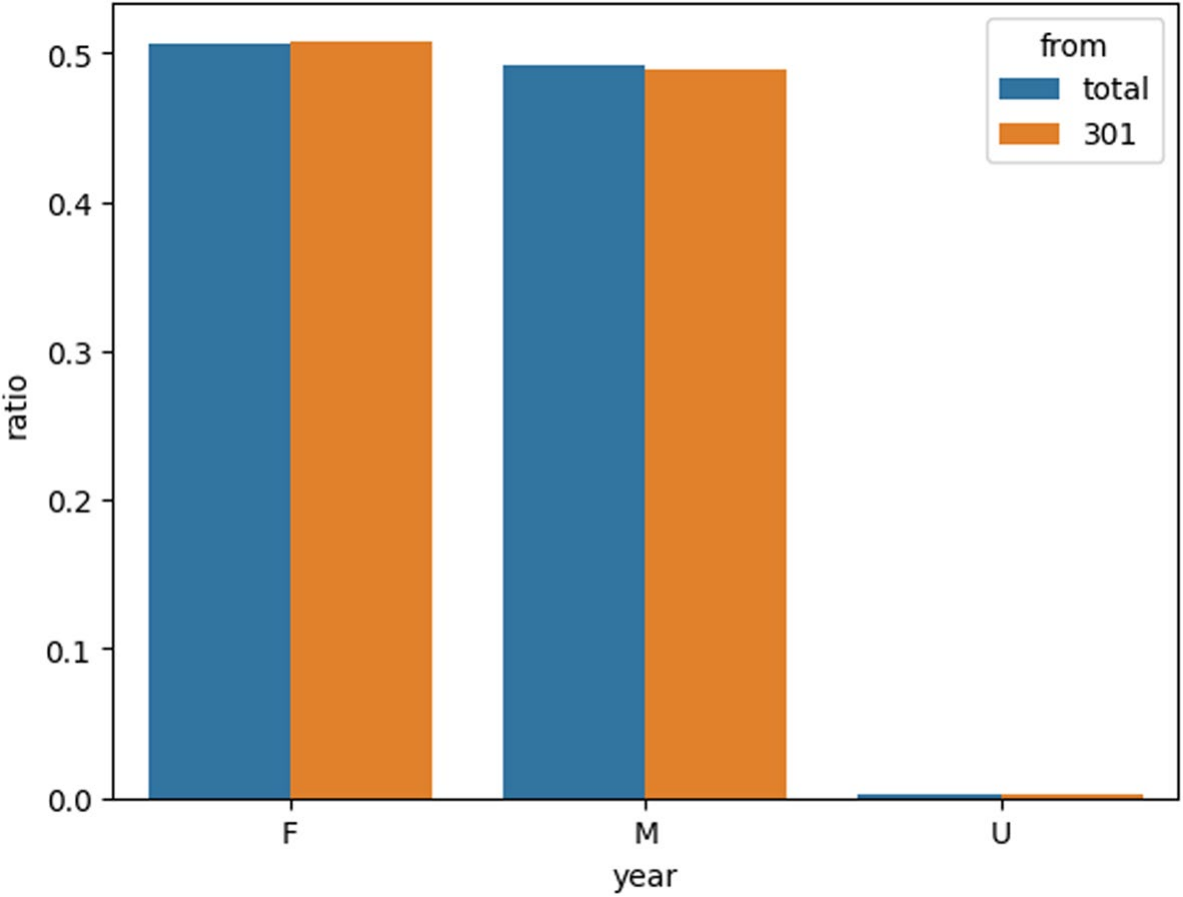
Gender ratio in NHI-CD-R301 and Full data of NHI-CD, illustrating that R301 has no significant difference in gender distribution from NHI-CD. U represents unknown gender

**Fig. 6:**
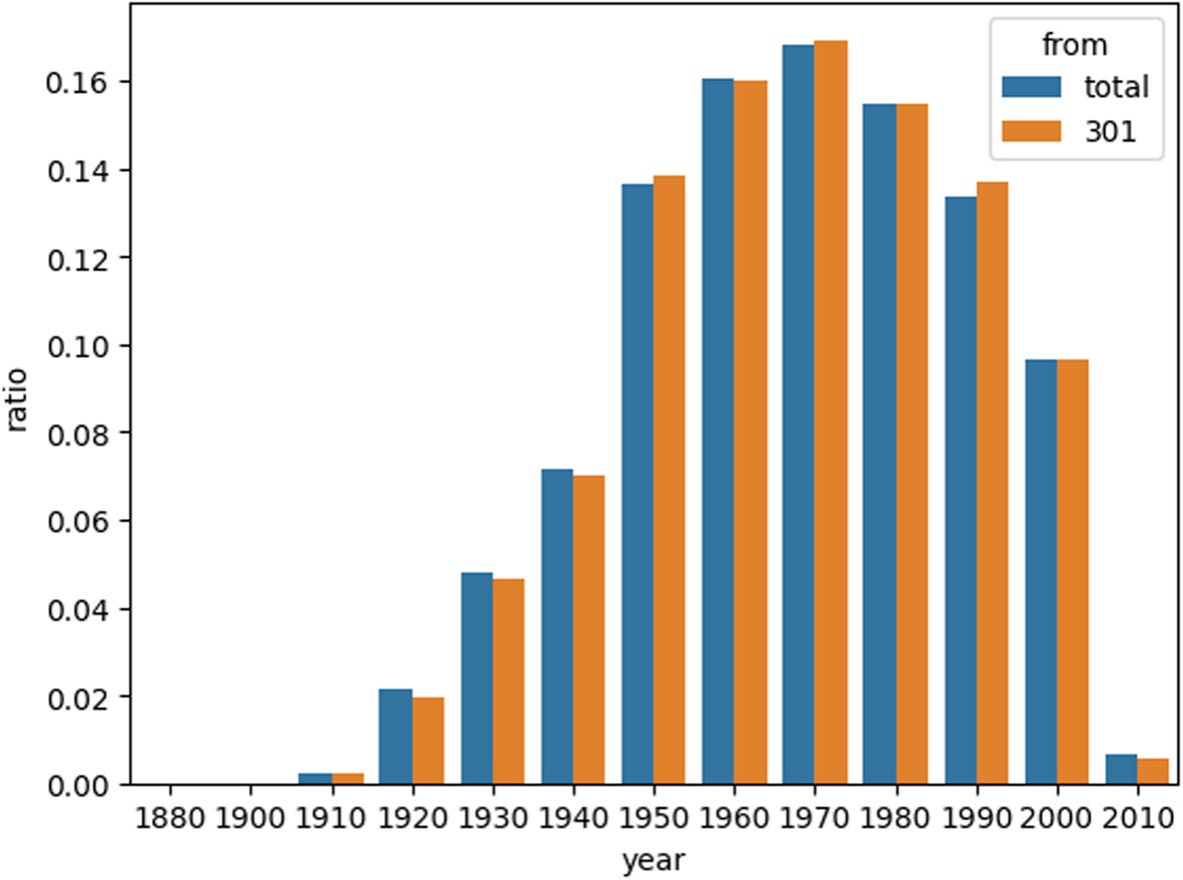
Age ratio in NHI-CD-R301 and Full data of NHI-CD, illustrating that R301 has no significant difference in age distribution from NHI-CD

NHI-CD is one subset of medical data collected by NHI. NHI collects health and medical records from all legal hospitals and clinics. NHI-CD extract records using a systematic sampling method on a monthly basis, together with the related records in details of ambulatory care orders (OO) from the Systematic Sampling CD.The dataset is double encrypted to protect patient privacy. Theoretically, it is impossible to track back to the patient, even for doctors. NHI-CD is commonly used in research by doctors in Taiwan (Figs. 5 and 6).

The NHI-CD dataset consists of around 1,000,000 randomly selected patients from the NHI collected records, which are updated every 5 years. To ensure efficient training of our model, these patients were randomly divided into 25 partitions, denoted as R301 to R325. Statistical analysis revealed no significant difference in the gender and age distribution of the patients in R301 (comprising 39,807 patients) and the full NHI-CD dataset (comprising 995,318 patients). Therefore, we selected R301 as our experimental dataset for further analysis and model development.

#### MIMIC-III

MIMIC-III (Medical Information Mart for Intensive Care III) is a large, freely accessible database of de-identified electronic health record data for patients who were admitted to critical care units at the Beth Israel Deaconess Medical Center between 2001 and 2012. It is commonly used for research purposes and has been a valuable resource for many studies in critical care, health informatics, and machine learning.

### ICD coding

Both NHI-CD and MIMIC-III contain Ambulatory care expenditures by visits, and each disease is encoded into the International Classification of Diseases, Ninth Revision, Clinical Modification (ICD-9-CM) codes^3^. According to guidelines provided by the National Health Insurance, the diseases recorded before 2006 follow the ICD-9-CM 1992 coding system, while those recorded after 2006 follow the ICD-9-CM 2001 coding system. While both MIMIC-III and NHI-CD are datasets from a single region, we do not consider variations from different regions. In our method, since we consider only the 4th digits of the diseases’ ICD9 codes, they remain the same across different versions in different years. Moreover, ICD-9-CM is completely comparable with the ICD-9. For brevity, we will refer to the codes as ICD-9 in the following. Compared with ICD-9, ICD-10 provides a more detailed classification system that allows for a greater specificity in coding medical conditions, which improves the ability to measure health care services, monitor public health, and conduct global epidemiological research. ICD-10 has a more complex structure and can accommodate a larger number of entries and more precise information. Since both ICD-9 and ICD-10 share similar structures, with disease categories remaining unchanged across different versions and there are also tools to convert ICD-9 to ICD-10, we believe that our model can be adaptable to fit the diseases with ICD-10 coding.

### Implementation

#### Noise disease set

**Fig. 7:**
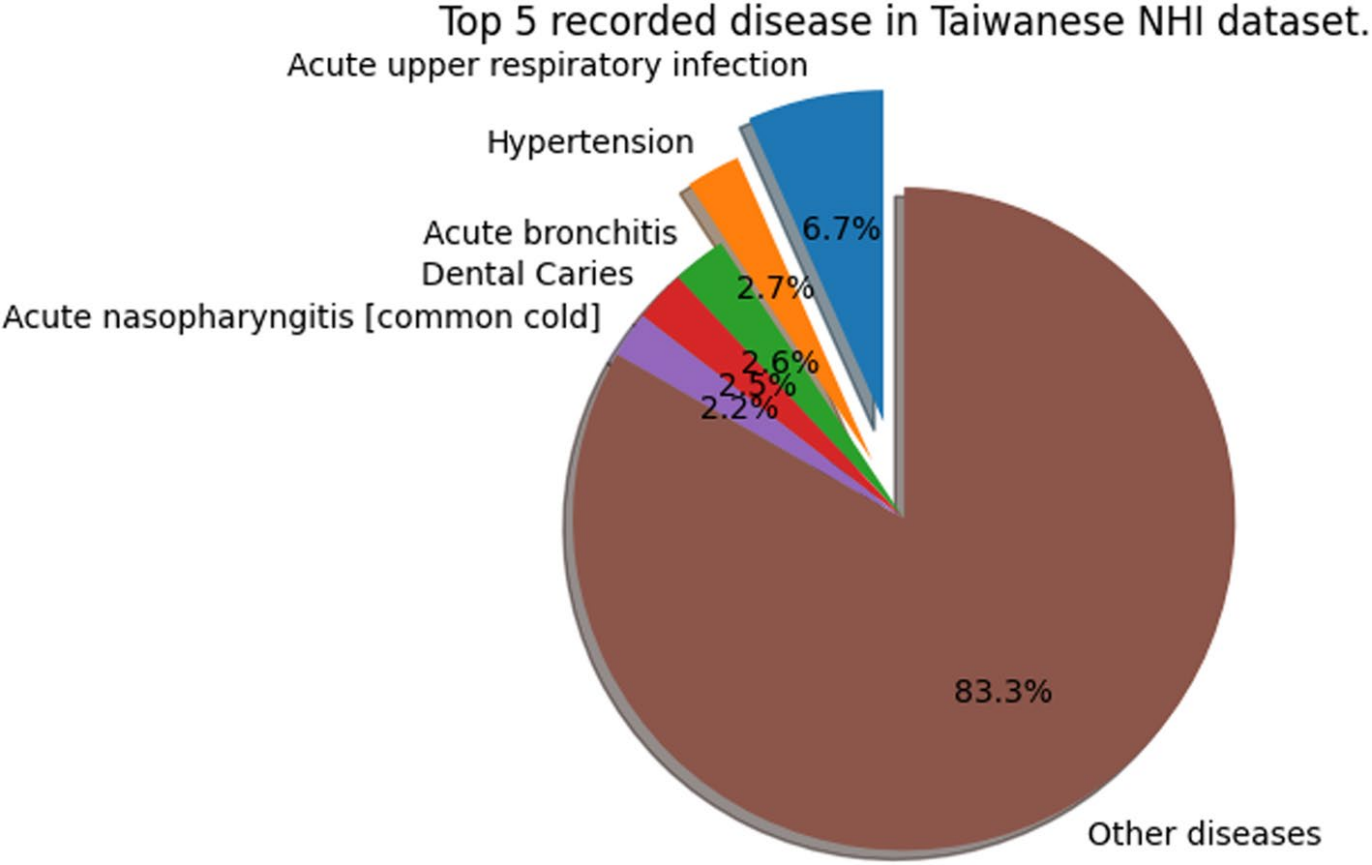
Top 5 diseases appearing in our dataset. Note that Acute upper respiratory infections, Acute bronchitis and Acute nasopharyngitis [common cold] are influenza-like diseases

Since al-BERT is inspired by the high frequency of recorded influenza-like illness cases within the NHI-CD dataset (Fig. 7), we chose influenza-like diseases which are defined in [3] as the noise disease set *S*. The disease in this set are listed in Table 3.

**Table 3:** ICD-9 codes and their name of diseases refer to influenza-like disease

ICD-9	Disease name
079.89	Other specified viral infection
079.99	Unspecified viral infection
460	Acute nasopharyngitis [common cold]
462	Acute pharyngitis
464.00	Acute laryngitis
464.10	Acute tracheitis
464.20	Acute laryngotracheitis
465.0	Acute laryngopharyngitis
465.8	Acute upper respiratory infections of other multiple sites
465.9	Acute upper respiratory infections of unspecified site
466.0	Acute bronchitis
466.11	Acute bronchiolitis due to respiratory syncytial virus (RSV)
466.19	Acute bronchiolitis due to other infectious organisms
478.9	Other and unspecified diseases of upper respiratory tract
480.0	Pneumonia due to adenovirus
480.1	Pneumonia due to respiratory syncytial virus
480.2	Pneumonia due to parainfluenza virus
480.8	Pneumonia due to other virus not elsewhere classified
480.9	Viral pneumonia, unspecified
484.8	Pneumonia in other infectious diseases classified elsewhere
485	Bronchopneumonia, organism unspecified
486	Pneumonia, organism unspecified
487.0	Influenza with pneumonia
487.1	Influenza with other respiratory manifestations
487.8	Influenza with other manifestations
490	Bronchitis, not specified as acute or chronic
780.6	Fever and other physiologic disturbances of temperature regulation
784.1	Throat pain
786.2	Swelling, mass, or lump in head and neck

#### Trigger and target disease set

In this scenario, our aim is to analyze the predictive influence of the prevalent disease, hypertension (401.9), on a patient’s health. We selected hypertension due to its higher prevalence in the dataset compared to acute upper respiratory infections (465.9), which represents a form of influenza-like disease. The next sentence prediction labels are determined by considering 401.9 as the trigger disease and selecting its top 6 related diseases as the target disease set. The target diseases include 402.9, 414.9, 250.0, 413.9, 272.4, and 401.1. The full names of these diseases can be found in Table 4. The disease information was extracted from the website http://www.icd9data.com/.

**Table 4:** ICD-9 codes and their name of the trigger diseases and target diseases we selected

ICD-9	Disease name
401.9	Unspecified essential hypertension
402.9	Unspecified hypertensive heart disease
414.9	Chronic ischemic heart disease, unspecified
250.0	Diabetes mellitus without mention of complication
413.9	Other and unspecified angina pectoris
272.4	Other and unspecified hyperlipidemia
401.1	Benign essential hypertension

#### Statistical difference of selected diseases

**Fig. 8:**
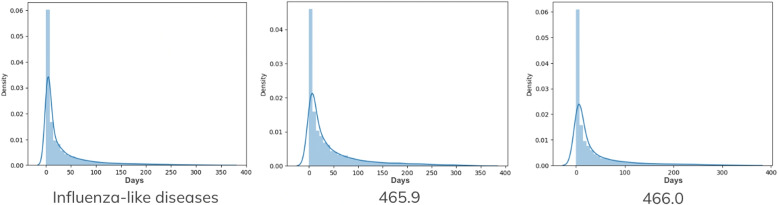
The x-axis represents the time intervals between occurrences of diseases within the dataset. Meanwhile, the y-axis displays statistical measures, offering insights into the occurrence patterns of diseases. Specifically, the statistical results pertain to diseases categorized as “influenza-like diseases”, including two specific examples in the dataset: 465.9 (Acute upper respiratory infections of unspecified site) and 466.0 (Acute bronchitis)

In this section, we discuss and provide reasons why we select the influenza-like diseases as noise diseases, and selected hypertension and its related diseases as the trigger and targets. As we predict and learn based on diagnosis records instead of data providing pharmacology and medical information, we observe the diagnose day interval of the same diseases. Figure 8 illustrates the statistical day intervals among all 27 influenza-like diseases and specifically for two diseases in the set, 465.9 and 401.9. The data show a typical pattern where diagnoses are often made within 10 days and exhibit only a single peak. This indicates that influenza-like diseases tend to be shorter-term diagnoses, occurring somewhat randomly over time.

**Fig. 9:**
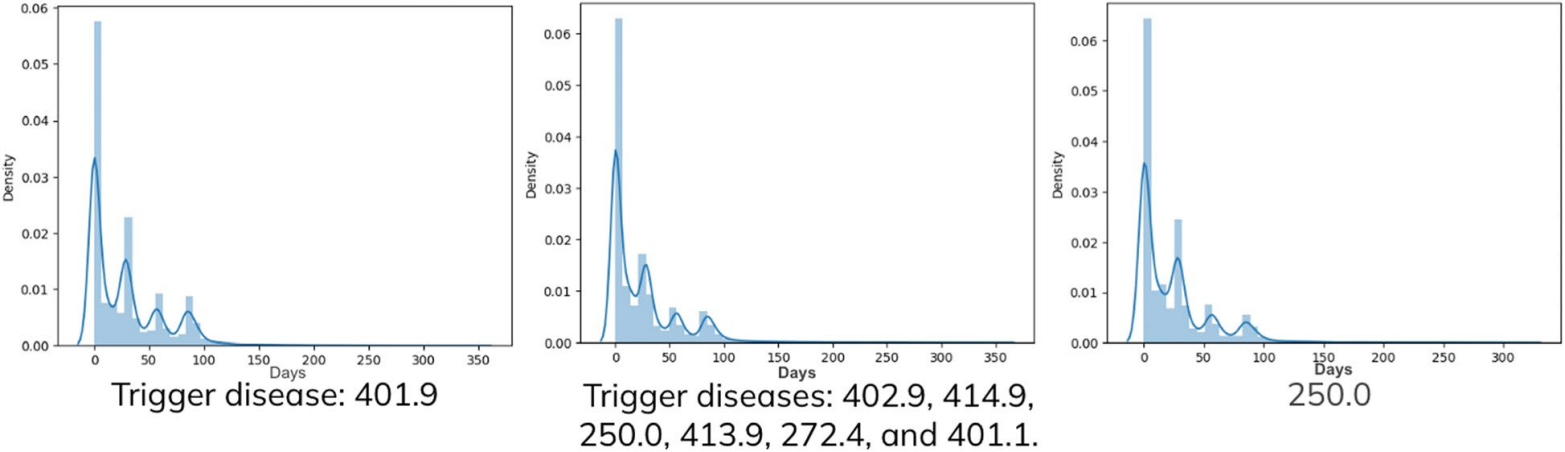
The x-axis indicates the intervals in days between instances of the same disease, while the y-axis illustrates statistical ratios or measures. The figure on the left-hand side displays the day interval statistical results specifically for disease code 401.9 (hypertension). The middle figure represents the results for all diseases within the target set, while the one on the right-hand side shows the statistical outcomes for a specific example within the target set, specifically disease code 250.0

On the contrary, Fig. 9 depicts the statistical day intervals between the trigger disease 401.9 and its corresponding target diseases. In this figure, it is noticeable that 401.9 and its related diseases exhibit similar characteristics. Their revisit day intervals show four distinct peaks. Figures 8 and 9 show the distinct patterns in the day intervals between diagnoses of specific diseases. While influenza-like diseases exhibit a single peak around a 10-day interval, suggesting shorter-term diagnoses that occur sporadically, the trigger disease 401.9 and its related diseases present a more structured pattern with multiple distinct peaks in their revisit day intervals.

#### Length of disease sequences

**Fig. 10:**
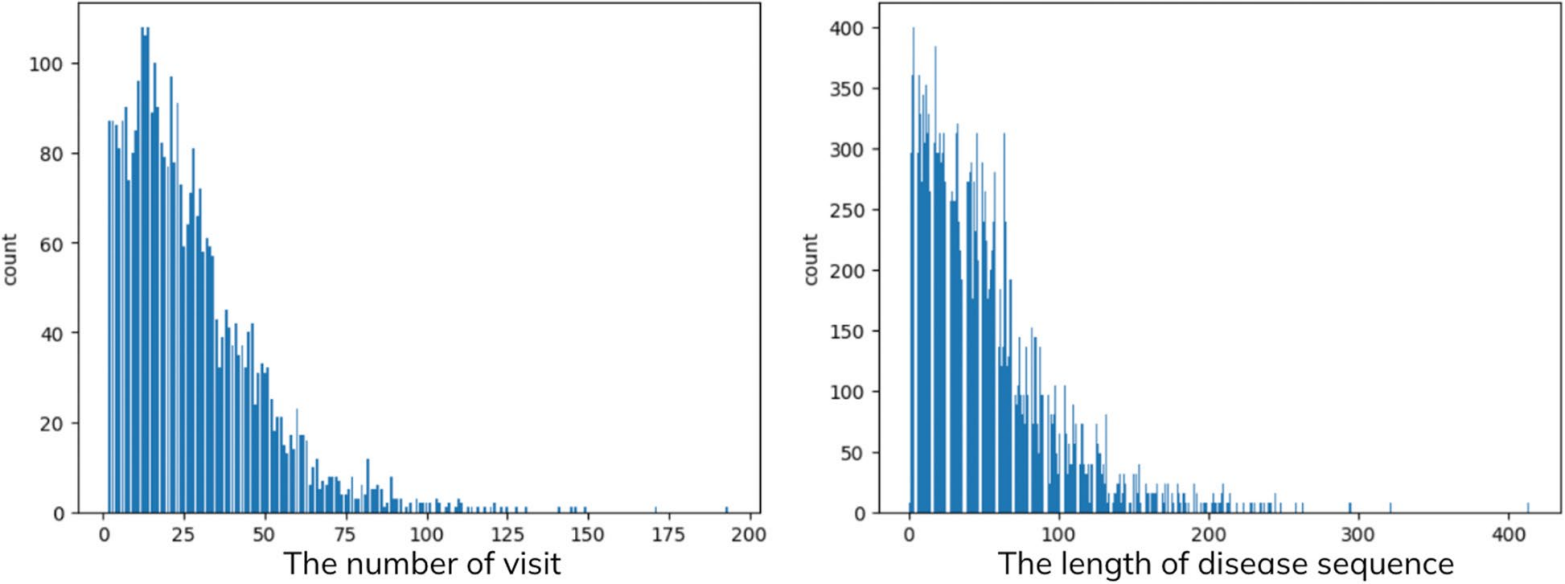
The chart on the left-hand side depicts the distribution of visit lengths, while the chart on the right-hand side illustrates the distribution of disease sequences in our training and testing datasets

Figure 10 displays the statistical distribution of both the number of visits per patient and the lengths of disease sequences. The data reveals that the average number of visits is 27.34 with a standard deviation of 20.93. In this study, each diagnosis can result in multiple disease codes, which collectively form disease sequences. The average length of these sequences is 51.45, accompanied by a standard deviation of 43.36. Based on these statistics, disease sequences shorter than the average length of 51 are padded with zeros until they reach this average length.

#### Adjustable parameter

The selection of adjustable parameters relies on the conditional probabilities in our dataset, as outlined in Table 5. It is notable that the probability of a diagnosis of influenza-like diseases is nearly equivalent to the probability when the patient is diagnosed with hypertension. Furthermore, all patients with hypertension also had at least a record of influenza-like disease diagnosis. We chose $\alpha = P(B|A)$ and $\beta = P(A|B)$.

**Table 5:** Set A comprises patients diagnosed with influenza-like disease, while set B consists of patients diagnosed with hypertension

Set	Probability
A	0.2053
B	0.9741
A | B	0.2107
B | A	1

Also, we set $k=2$ and time $T=1$ year. These settings are referred to in [26, 30].

### Baselines

For the baselines, we explore various learning modules and compare them to our proposed al-BERT model.

#### Overall model

In this experiment, we compare the following disease prediction models.

- **original Med-BERT** [1]: Apply BERT by preprocessing medical records to sentence-like structures. Medical records include disease descriptions, drugs, and procedures. These records are arranged by time and medical priority, then consider codes as words and arranged records as sentences.
- **retain-BERT**: In this scenario, we utilize RETAIN as a base recurrent neural network preceding BERT. Similar to our learning module, we utilize RETAIN to extract weights for each disease after the RNN layer, filtering the diseases based on the weights it learns.
- **Random-BERT**: Randomly delete the located disease (flu) in the diagnose sequence. Each located disease has a 30% chance of been deleted. Our goal for this pattern is to compare the performance of training with the original disease sequence and training with a shorter disease sequence. We take 30% as filter chances according to the ratio of influenza-like diseases and injuries in the dataset.

#### Ablation study: without BERT

We compare our attention learning (al) with LSTM [31] and RETAIN [15] without connecting to BERT.

- **LSTM**: LSTM is employed to determine whether to filter each record. LSTM’s ability to capture sequential dependencies makes it suitable for learning the relevance of individual records in the context of the overall sequence.
- **RETAIN w/o BERT**: RETAIN is a reverse time attention model using two RNN layers. These RNN layers are for visit-level attention and variable-level attention. Using the generated attentions, we obtain a context vector *c* which represents a weight of visits. We extract vector *c* as weights to adjust the importance of each visit.
- **Double attention (Ours) w/o BERT**: We execute all the blocks before the extraction of diseases. Specifically, our model comprises two layers of attention: the initial layer operates in parallel, learning the significance of the entire sequence and selected subsequences, while the second layer processes the overall combined sequence from the previous layer.

### Metrics

To measure the prediction quality, we use accuracy, precision, recall (sensitivity), f1 and ROC-AUC score. Recall is regarded as the most important for medical studies in the above-mentioned metric, since high recall means missing fewer positive results [32].

### Experiment result

#### Overall model

For the BERT-based models, we compare their quality in masked language model tasks and next sentence prediction tasks. Table 6 shows the masked language model and next sentence prediction accuracy. We extend the result in the experiment for the learning module part. During the fine-tuning stage, patients who had been diagnosed with the trigger disease 401.9 (Hypertension) were selected, and a [SEP] token was inserted afterwards to determine if any of the diseases in the target disease set were diagnosed. A total of 2,691 patients were selected, and the maximum disease sequence length shown in Table 6 was set to 75.

**Table 6:** Result of each method concatenating with our pretrained BERT. MLM stands for masked language model and NSP stands for next sentence prediction. In this table, the input disease sequence length is set to 75. The first five metric (AUC-ROC, accuracy, precision, recall, F1) represent the task of predicting the appearence of the target disease

Model	AUC-ROC	Accuracy	Precision	Recall	f1	MLM	NSP
Med-BERT	0.9282	0.8591	0.8348	0.6329	0.7139	0.5375	0.8650
Random-BERT	0.9228	0.8523	0.7985	0.5894	0.6893	0.5419	0.7925
RETAIN-BERT	0.8508	0.7866	0.6781	0.6812	0.6395	**0.5634**	0.7975
al-BERT	**0.9383**	**0.8725**	**0.8450**	**0.7874**	**0.7743**	0.5626	**0.8675**

The bold case represents the best result under a single metric

The rationale behind selecting a disease length of 75 is rooted in practical considerations. This choice is derived from the median length of a patient’s records in a year, which is 25, with each record containing a maximum of 3 diseases. Flattening these records results in a length of 75. This selection strikes a balance by capturing sufficient information while avoiding excessive zero padding during BERT model training. However, for a comprehensive evaluation of the model’s performance, we also compared results using a longer disease length of 285, which is 95 records flattened by 3 diseases. This length represents the 99th percentile of the longest patient records across all data in the NHI-CD-R301 dataset.

**Table 7:** The masked language model and next sentence prediction result in NHI-CD-R301 for disease sequence length 285

Model	AUC-ROC	Accuracy	Precision	Recall	F1	MLM	NSP
retain-BERT	0.6789	0.6176	0.6197	0.9022	0.6933	0.4669	0.9225
al-BERT	0.7889	0.7056	0.7489	0.7597	0.7109	0.7767	0.8950

Table 7 presents the comparison of masked language model performance using retain-BERT with different disease sequence lengths. These results indicate that our attention-based learning module efficiently captures essential information for understanding a disease, leading to improved performance compared to retain-BERT. However, al-BERT encountered challenges in the next sentence prediction task. We attribute this to a defect in the learning module, where target diseases have a higher likelihood of being filtered out in longer sequences, thereby influencing the decision-making process of al-BERT.

Notice that in both Tables 6 and 7, our model outperforms the original Med-BERT in the masked language task. Particularly, the combination of random selection and Med-BERT achieves the highest performance, while the original Med-BERT, which does not filter out any diseases, achieves only 38.15% accuracy. This observation suggests that feeding shorter disease sequences into the BERT-based model enables it to perform better in masked language tasks by reducing the number of decisions that need to be made.

### Ablation study

Further, we compare the prediction results without the BERT module. Table 8 shows the result of different methods to capture essential diseases. In this experiment, we pretrained the BERT model by using NHI-CD-R301 data.

**Table 8:** Comparing different learning modules

Module	AUC-ROC	Accuracy	Precision	Recall	f1
LSTM	0.8018	0.7409	0.5389	0.4686	0.5013
RETAIN	0.9080	0.8376	0.7979	0.5652	0.6592
ours(al)	**0.9181**	**0.8550**	**0.8134**	**0.7585**	**0.7441**

The bold case represents the best result under a single metric

Our method achieved the highest performance among the listed methods, with an accuracy of 0.8550, AUC-ROC of 0.9181, precision 0.8134, recall 0.7585 and f1 score of 0.7441. Based on these results, it appears that our attention learning module outperforms the other methods in terms of these metrics. Moreover, domain experts claim the importance in recall value; our recall score is significantly higher than that of other methods.

**Table 9:** Comparison of methods without BERT. We use MIMIC-III in this experiment

Model	AUC-ROC	Accuracy	Precision	Recall	F1
LSTM	0.9247	0.8918	0.8754	0.9701	0.9204
RETAIN	0.9505	0.8826	0.9371	0.8846	0.8812
al (ours)	**0.9621**	**0.9280**	**0.9448**	**0.9769**	**0.9304**

The bold case represents the best result under a single metric

Table 9 provides a comparison of non-BERT-based disease prediction models, including LSTM, RETAIN, and a part of our proposed model (referred to as al, for attention learning). The experiment was conducted using the MIMIC-III dataset.

In terms of performance metrics, our attention learning outperforms both the LSTM and RETAIN models in terms of AUC-ROC, accuracy, precision, recall, and F1 score. Specifically, our attention learning method achieved AUC-ROC of 0.9621, demonstrating its ability to capture disease patterns. It also achieved accuracy of 0.9280, demonstrating its ability to correctly classify disease cases. Moreover, our model achieved high precision (0.9448) and recall (0.9769), indicating its capability to accurately identify true positives and minimize false negatives.

### Case study

**Fig. 11:**
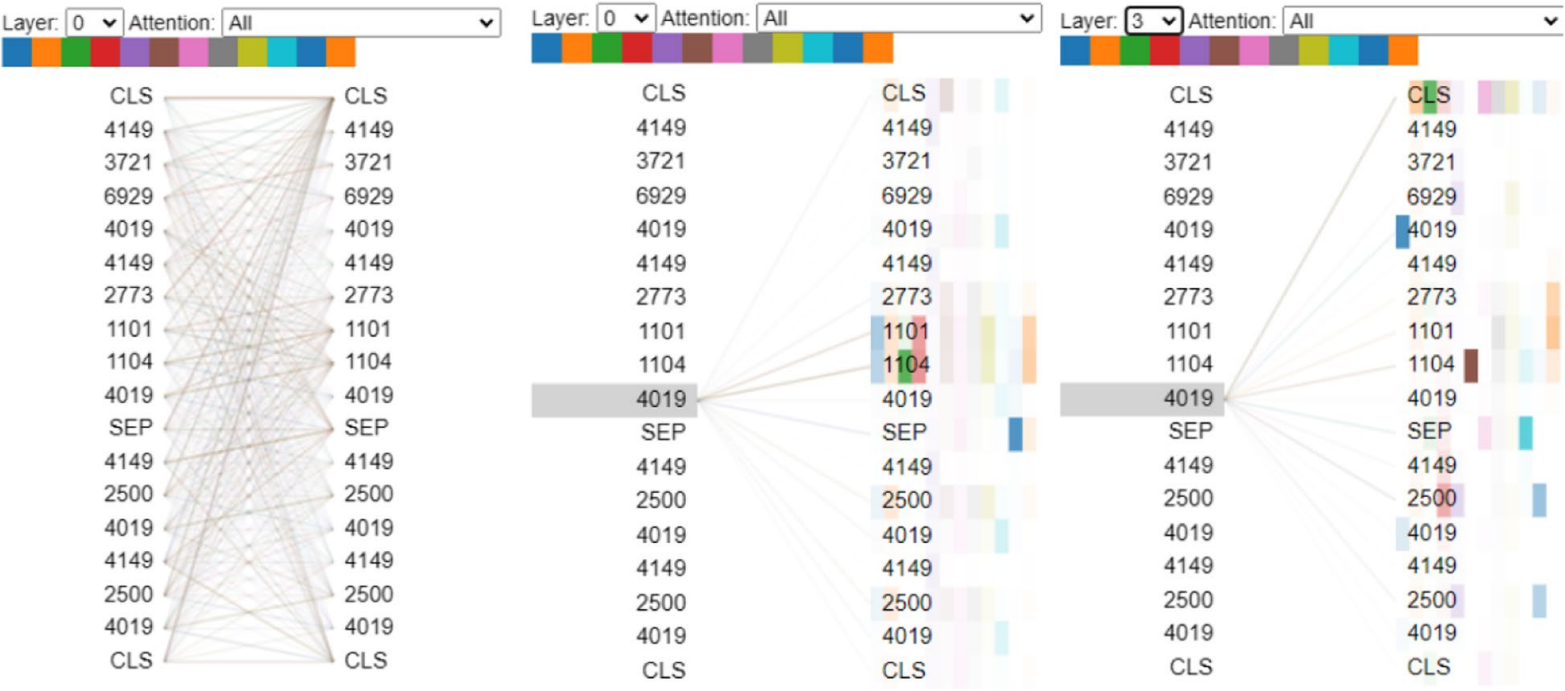
This figure shows the visualization results for a patient predicted to develop 2500 (diabetes) in the future. Notably, focusing on diseases after [SEP], some diseases exhibit strong connections (indicated by deeper color). Specifically, in layer 3, the connection between 4019 and 2500 appears stronger than with other diseases after [SEP]

This case study demonstrates the application of our al-BERT model to predict the likelihood of a patient developing diabetes by visualizing a truncated sequence of EMR data using bertviz.We visualize our results using bertviz [33]. Since the original experiment of al-BERT had a sequence length of 75, which would be too long for visualization comprehension, we have captured a subsequence of length 15 to illustrate an example here. Figure 11 illustrates an example to use next sentence prediction task of al-BERT to predict whether the patient will develop diabetes (2500) in the future. In the left of Fig. 11, we display the full dependency connections between their records and select 4019 (hypertension) to highlight its significance for our prediction.

We can see that the second occurrence of 4019 has a strong connection with some diseases after the [SEP] token, especially with 2500 on layer 3. In this case, these two disease sequence are likely to be the next sequence, therefore we predict the label as 1, which means this patients are more likely to have 2500 in the future.

## Conclusion

We proposed a model, al-BERT, a BERT-based model for disease prediction that incorporates domain knowledge, such as comorbidity and the distance between diseases. Our model was designed to address the disease prediction problem by applying a semi-supervised attention learning method and BERT. It learns from a patient’s diagnosis records, undergoing preprocessing via bidirectional RNN to comprehend their characteristics. Concurrently, a parallel reverse RNN aids in identifying and capturing noise diseases. The resulting information is then combined and processed through a simple RNN once more, extracting a subsequence that best represents the patient’s more significant medical history. After extracting this subsequence, it is considered as sentences, and the BERT model is trained using masked language modeling and next sentence prediction tasks. Through experiments and evaluations on real-world datasets, our model has demonstrated improved performance compared to other baselines. The combination of our attention learning module and pretrained BERT has outstanding results in accuracy, AUC-ROC, and F1 scores in capturing essential diseases and predicting future diseases.

Since the latest datasets are based on ICD-10, we also aim to conduct experiments using ICD-10 in the future. ICD-10 and ICD-9 are constructed in the same manner [34] but provide more detailed information, which could be more closely aligned with a language representation. Therefore, we anticipate that our work will yield impressive results.

al-BERT effectively integrates domain knowledge, applies attention mechanisms, and leverages the power of pretrained BERT to improve disease prediction. The model shows promise in capturing essential information from disease records, and can be applied to real-world scenarios for predicting future diseases. The increasing popularity of large language models (LLMs) opens up possibilities for applying more information within Electronic Medical Records (EMRs); these potentials remain a subject for future work.

## Data Availability

NHI-CD (https://nhird.nhri.edu.tw/) belongs to National Health Insurance Research Database, Taiwan. This study is based in part on data from the National Health Insurance Research Database provided by the National Health Insurance Administration, Ministry of Health and Welfare and managed by National Health Research Institutes in Taiwan. The interpretation and conclusions contained herein do not represent those of National Health Insurance Administration, Ministry of Health and Welfare or National Health Research Institutes. MIMIC-III (https://physionet.org/content/mimiciii/1.4/) is available on PhysioNet, only credentialed users who sign the DUA can access the files. For more information: Johnson, A., Pollard, T., & Mark, R. (2016). MIMIC-III Clinical Database (version 1.4). PhysioNet. https://doi.org/10.13026/C2XW26.

## Footnotes

1 https://www.cdc.gov/nchs/icd/icd9cm.htm

2 We design the edge weights by extending Yang’s methodology, which considers shared comorbidities, the frequency of concurrent disease occurrences, and Shannon entropy. Interested readers are encouraged to refer to their work [26] for more detailed information.

3 https://www.cdc.gov/nchs/icd/icd9cm.htm
